# Characterization of Microbiome on Feces, Blood and Milk in Dairy Cows with Different Milk Leucocyte Pattern

**DOI:** 10.3390/ani11051463

**Published:** 2021-05-19

**Authors:** Elisa Scarsella, Alfonso Zecconi, Michela Cintio, Bruno Stefanon

**Affiliations:** 1 Department of Agriculture, Food, Environmental and Animal Science, University of Udine, 33100 Udine, Italy; scarsella.elisa@spes.uniud.it (E.S.); michela.cintio@uniud.it (M.C.); 2 Department of Biomedical, Surgical and Dental Sciences–One Health Unit, University of Milan, 20100 Milan, Italy; alfonso.zecconi@unimi.it

**Keywords:** microbiome, mastitis, bovine, entero-mammary pathway

## Abstract

**Simple Summary:**

Mastitis is an inflammation of the mammary gland caused by microorganisms and associated with an altered immune response. Recently, several studies hypothesized that a translocation of some bacteria from the gastrointestinal tract to the mammary gland can occur and that this bacterial crossing could be the cause of certain mastitis. The aim of this research is to investigate the bacteria translocation from the gut to the mammary gland, the so-called entero-mammary pathway, through the study of the fecal, blood and milk microbiome. Cows were recruited on the basis of their mammary gland health status and classified as healthy, at risk of mastitis and with mastitis. The microbial composition of feces, blood and milk were analyzed through high-throughput sequencing technique and the results were checked through a quantitative real-time PCR analysis. Although small differences were found in the microbiome of these three specimens between the groups of animals, beta biodiversity, that is, the ratio between whole and individual species diversity, highlighted a microbial community change in the milk of cows with different udder health conditions. The three matrices shared a high number of taxa; however, our results do not confirm a bacterial crossing from gut to milk, that still remains hypothetical.

**Abstract:**

Mastitis is an inflammatory disease of the mammary gland, caused by the invasion of microorganism on this site, associated with an altered immune response. Recent studies in this field hypothesize that the origin of these pathogens can also be from the gastrointestinal tract, through the entero-mammary pathway in relation to an increase in gut permeability. In this study, we wanted to investigate if inflammatory status of the mammary gland is related to an alteration of gut permeability. The microbiome of feces, blood and milk of lactating cows, recruited on the basis of the total somatic cell count and of the percentage of polymorphonuclear neutrophils and lymphocytes, was studied. Cows were divided into healthy (G), at risk of mastitis (Y) and with mastitis (R) classifications. The bacterial DNA was extracted and the V3 and V4 regions of 16S rRNA sequenced. Moreover, the quantification of total bacteria was performed with quantitative real-time PCR. A non-parametric Kruskal–Wallis test was applied at the phylum, family and genera levels and beta biodiversity was evaluated with the unweighted UniFrac distance metric. Significant differences between groups were found for the microbial composition of feces (Clostridiaceae, Turicibacteriaceae for family level and *Clostridium*, *Dorea*, *SMB53* and *Turicibacter* for genus level), blood (Tenericutes for phylum level and *Mycoplasma* for genus level) and milk (OD1 and Proteobacteria for phylum level, Enterobacteriaceae and Moraxallaceae for family level and *Olsenella* and *Rhodococcus* for genus level). The beta biodiversity of feces and blood did not change between groups. Significant differences (*p* < 0.05) were observed between the beta diversity in milk of G group and Y group and between Y group and R group. The number of taxa in common between feces, blood and milk were 8 at a phylum, 19 at a family and 15 at a genus level. From these results, the bacterial crossing from gut to milk in cows was not confirmed but remained hypothetical and deserves further investigation.

## 1. Introduction

Recent studies suggest that mammary epithelial cells have a role in mammary gland defense [1,2]. Mammary epithelial cells are highly responsive or affected by bacteria and metabolites derived by a leaky gut condition (Rodriguez, 2014) [3]. However, the first defense line against infection in the udder is usually considered the innate immune system [4], and recent analytical advances allow one to measure in milk not only the total somatic cell count (SCC) but also the percentage of polymorphonuclear neutrophils and lymphocytes [5,6]. A recent study showed the mounting of an inflammatory process leads to an increase in the overall amount of polymorphonuclear neutrophils and lymphocytes (P + L) of 1 log, from 10^9^ to 10^10^ in the milk and the assessment of the total amount of these cells is a benchmark for studies on udder immune response [7]. Differential somatic cell count (DSCC) is a complementary parameter also useful to differentiate chronic from acute mastitis and healthy cows. DSCC is the percentage of neutrophils plus lymphocytes to total SCC [6,8]. High values of this parameter indicate the onset or the presence of an inflammatory response.

The development of mastitis is due to the invasion of microorganisms into the mammary gland associated with an altered immune response [9]. Opportunistic and pathogen bacteria, as well as environmental pathogens, can invade the teat duct and progressively colonize mammary gland. However, some studies have hypothesized that microbes and their metabolites in the udder can have also an intestinal origin [10,11].

Enteric cells are continuously exposed to feed and microbial antigens [12] and the local immune system is able to differentiate commensal from pathogenic microorganisms, mounting an immune response against the latter. In the gut, the recognition of “beneficial” or “harmful” microorganisms is due to the presence of dendritic cells [13], but environmental factors can shift commensal to pathogenic bacteria, causing dysbiosis, gut inflammatory response and eventually a disruption of the gut tight junction [14]. One important factor regulating the interplay between the gut microbiome and host is the maintenance of the integrity of the intestinal mucosa [15]. The disruption of this integrity is known as “leaky gut” and in dairy cows it has been reported that dysfunction of the intestinal barrier causes inflammation, affects metabolism and reduces productivity in lactating Holstein cows [15]. In this situation, an invasion of microbes into the host can occur, mainly through the blood stream and lymphatic system [16], especially in ruminants that are naturally exposed to a rich gut microbiome ecosystem. When the invasion is concomitant to a reduction in immune surveillance, bacteria can spread to other organs, among these the mammary gland [17]. When this situation occurs, live microbes are detected in the bloodstream, and some evidence in animals [18,19] and humans [20,21,22] has confirmed the presence of cultivable bacteria in the blood in subjects without sepsis. Although the origin of these bacteria is mainly attributed to the translocation from the gastro-intestinal tract [23], it has been also suggested that the oral cavity and skin can contribute to diffuse the microbial population into the blood [24]. It is hypothesized that bacteria found in healthy human blood may be in a dormant state [25] or are present in their L-forms [26], thus not causing infectious disease.

In view of these considerations, in the present research, we wanted to investigate if the inflammatory status of the mammary gland, assessed with SCC and DSCC parameters, associated with environmental mastitis, can be potentially related to a bacteria translocation from the gut to the mammary gland. For this aim, the composition of the microbial community in feces, blood and milk of lactating cows was assessed to investigate if a translocation of bacteria from the gut to the udder could occur.

## 2. Materials and Methods

### 2.1. Animals and Housing

Sixty Holstein Friesian cows from a herd of 140 lactating cows housed in a local farm (46.1134059, 13.2817455; N 46°6′48.261″, E 13°16′54.283″; Italy) were recruited for this study. The sampling collection was performed directly to the local farm. The cows were preselected based on SCC and DSCC values of the previous monthly official record of the Breeder Association (Associazione Allevatori del Friuli Venezia Giulia, Codroipo, Italy; www.aafvg.it, accessed on 27 April 2021). The cows of the herd were divided in 4 groups according to the already existing classification [27,28] in healthy (group G, SCC/mL < 200,000 and DSCC ≤ 69.3%), at risk (group Y, SCC/mL < 200,000 and DSCC > 69.3%), chronic (group O, SCC/mL > 200,000 and DSCC ≤ 69.3%) and with subclinical mastitis (group R, SCC/mL > 200,000 and DSCC > 69.3%). However, with 200,000 SSC being considered a threshold for mastitis [27,29,30], groups O and R were considered to be equally affected and grouped in the single group R. Based on the SCC and DSCC data measured the month before sampling, 60 cows (20 for each group) were identified. The milk was then collected for analysis the following month, in correspondence to the next official record. On the basis of the new measured values of SCC and DSCC, 34 cows resulted healthy and were assigned to group G (11 primiparous and 23 pluriparous; 10 days in milk (DIM) < 70 and 24 DIM > 70), 13 cows were at risk of mastitis and assigned to group Y (6 primiparous and 7 pluriparous; 2 DIM < 70 and 11 DIM > 70) and 13 cows were affected and assigned to group R (2 primiparous and 11 pluriparous; 3 DIM < 70 and 10 DIM > 70). The animals were housed in free stalls with cubicles, were fed with the same total mixed ration (Table S1) and were not treated with antibiotic since the last 20 days before the sampling collection day. The milking parlor (parallel 12 + 12) was adjacent to the barn. All protocols, procedures and the care of the animals complied with the Italian legislation on animal care and were evaluated and approved by the bioethical committee of the University of Udine (OPBA, #9/2020).

### 2.2. Collection of Samples

Feces, blood and milk samples were collected during the evening milking. Briefly, after discharging the first flow of milk from each quarter, the teats were disinfected by dipping in iodine tincture. Disposable wipes and disinfection with ethanol 70% was applied to clean each quarter. For each cow, about 50 mL of milk, (a mixture including all the four quarters) was sampled in sterile Falcon tubes immersed on ice during the collecting period. Whole blood was collected in a K_3_-EDTA tube (Sarstedt, Nümbrecht, Germany). with venipuncture from the radial vein after shaving the coat and careful sterilization. A grab sample of fecal material was transferred to sterile gloves into a sterile container. Each sample was immediately frozen at −20 °C when it arrived at the laboratory and stored until the analysis.

### 2.3. DNA Extraction, Library Preparation, Sequencing and Taxonomic Annotation

Microbial DNA was extracted following the instructions with two commercial kits, based on the starting material. DNA from fecal samples was extracted from 150 mg of starting material using a Fecal DNA MiniPrep kit with a bead beating step (Zymo Research; Irvine, CA, US). Microbial DNA from milk samples was extracted from 250 μL of the starting material using the same Fecal DNA Miniprep kit, with the addition of a preliminary warming step of 10′ at 70 °C [31] followed by a bead lysis process. DNA from blood samples was extracted from 200 μL of the starting material using a Exgene™ Clinic SV kit (GenAll Biotechnology, Seoul, Korea). A ZymoBIOMICS™ Microbial Community Standard (Zymo Research, Irvine, CA, USA) was used as an internal control to assess the reproducibility of the entire pipeline, from the DNA extraction method to taxonomic annotation. The mock community contained eight bacterial species: *Pseudomonas aeruginosa* (4.2%), *Escherichia coli* (10.1%), *Salmonella enterica* (10.4%), *Lactobacillus fermentum* (18.4%), *Enterococcus faecalis* (9.9%), *Staphylococcus aureus* (15.5%), *Listeria monocytogenes* (14.1%) and *Bacillus subtilis* (17.4%). The expected composition of the mock community was certified by the manufacturer. DNA concentration was measured with a QubitTM 3 Fluorometer (Thermo Scientific; Waltham, MA, USA) and the 16S rRNA of V3 and V4 regions amplified for library preparation, also adding the indexes for sequencing, using a Nextera DNA Library Prep kit (Illumina; San Diego, CA, USA), following manufacturer’s instructions, freely accessible through the Illumina website (www.illumina.com, accessed on 27 April 2021) and primers [32]. The resulting amplicons were sequenced with a NovaSeq6000 (Illumina; San Diego, CA, USA) in 2 × 250 paired-end mode, following the standard procedures. QIIME2 suite (Quantitative Insights Into Microbial Ecology) was used to process the raw sequences [33], which were uploaded to NCBI Sequence Read Archive (Bioproject ID: PRJNA725200). After demultiplexing, sequenced reads were merged and denoised through DADA2 process (referenza dada2) and the reads that passed the quality check (Phred score ≥ 30) were annotated for 16S rRNA against the most recent Greengenes database (version gg.13_8.otus.tar.gz), with 99% identification with reference sequences. Chimeras were also detected and then filtered from the reads and the remaining sequences were denoised into amplicon sequence variants (ASVs) by using an open reference approach of QIIME 2.

### 2.4. Quantitative Real-Time PCR (qPCR)

For the quantification of total bacteria, qPCR using the oligonucleotides tested by AlShawaqfeh et al. [34] was used and the ZymoBIOMICS™ Microbial Community Standard was used as a DNA positive control for the quantification of the total 16S copies DNA/g bacteria. For the SYBR-based qPCR assays the protocol reported by AlShawaqfeh et al. [34] was applied, with some modifications. SYBR-based reaction mixtures (total 12.5 μL) contained 6.25 μL of Platinum™ SYBR™ Green qPCR SuperMix-UDG (Invitrogen, Carlsbad, CA, USA), 3.25 μL of water, 0.25 μL of each primer (final concentration: 300 nM), and 2.5 μL of DNA previously diluted at 1 ng/μL. PCR conditions were 95 °C for 2 min, and 40 cycles at 95 °C for 5 and 10 s at the optimized annealing temperature. A melt curve analysis was performed for SYBR-based qPCR assays under the following conditions: 1 min at 95 °C, 1 min at 55 °C, and 80 cycles of 0.5 °C increments (10 s each). A CFX96 Touch System (Bio-Rad Laboratories, Hercules, CA, USA) was used for all qPCR assays. All samples were run in triplicate and data were expressed as average values and standard deviations.

### 2.5. Statistical Analysis

Cows were classified on the basis of SCC and DSCC, as reported above. The annotated sequences of each sample were normalized to ‰ abundance profiles for each taxonomic level in relation to the total number of reads of each sample and referred to as relative abundance (RA). Taxa with RAs lower than 1‰ in more than 30 samples were not considered for the statistical analysis. RAs were transformed into absolute abundances (AAs), multiplying each data with the quantification of total bacteria revealed by the qPCR for each sample [35]. A Shapiro–Wilk test was initially applied to check the normality of the variable distribution. Since microbiota data deviated from normality, a non-parametric Kruskal–Wallis test was applied at the phylum, family and genera level of fecal, milk and blood samples. The Bonferroni correction for multiple comparison was applied as a post hoc test to determine differences in means. The Kruskal–Wallis test was performed with XLSTAT [36]. Beta biodiversity was evaluated with the phylogeny based on unweighted UniFrac [37] distance metric and visualized using a principal coordinate analysis (PCoA) plot. The association between microbiome composition and covariate (classification of cows in the groups G, R and Y) was tested using PERMANOVA. The significance of the PERMANOVA test was determined using 999 permutations with adjustment for multiple testing. Statistical analyses were performed with bioinformatic pipelines available through QIIME2. A *p*-value below 0.05 was considered statistically significant.

## 3. Results

The study was conducted in one farm and the cows were recruited based on their monthly official record. The main objective of this experiment was to compare the microbial population of different compartments, such as feces, blood and milk, on cows differing for SCC and DSCC. The description of the cows and how they were divided is reported in Table 1. After the second monthly milk record, the resulting groups were unevenly distributed between primiparous and multiparous with only few cows in some classes. Milk yield was equal to 35.1 kg (SD: 12.4 kg), 35.9 kg (SD: 12.2 kg) and 39.1 kg (SD: 8.4 kg) for the G, Y and R groups, respectively (*p* > 0.05). Since the milk production was not significant in the three groups, it has not been included for the statistical analysis.

**Figure 1:**
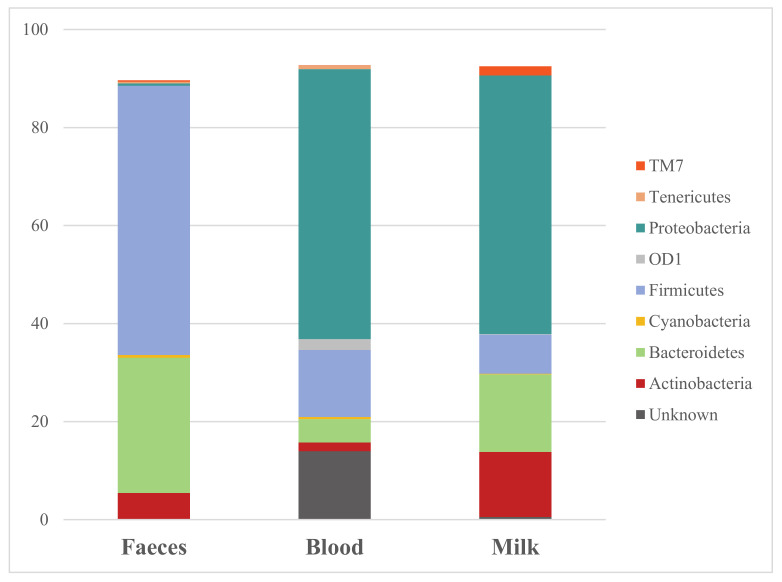
Microbiome composition at a phylum taxonomic level in feces, blood and milk sampled from lactating cows of all groups.

The bacterial composition measured in feces, blood and milk showed differences in terms of abundances and taxonomic annotations. In Figure 1, the mean of the phyla abundances shared by the three specimens were reported. The three most abundant phyla in feces were Firmicutes (54.93%), Bacteroidetes (27.57%) and Actinobacteria (5.36%). The other phyla showed in Figure 1, Cyanobacteria (0.56%), Proteobacteria (0.47%) and Tenericutes (0.33%), were presented in a very low abundance. Proteobacteria was the most abundant phylum (55.09%) in blood, followed by Firmicutes (13.68%) and Bacteroidetes (4.77%). The abundances of the other phyla decrease substantially and among the phyla OD1 (2.19%), Actinobacteria (1.78%) and Tenericutes (0.78%) were detected. A large percentage of bacteria were not assigned in blood samples (13.97%). In milk, the most abundant phylum was Proteobacteria (52.75%), followed by Bacteroidetes (15.85%) and Actinobacteria (13.25%). In these samples, bacteria belonging to the Firmicutes phylum were only the 7.97% and it was also found the taxa TM7 with 1.90% and OD1 with 0.13%. Of note, in milk samples, the phylum Tenericutes was not reported. All the data reported until now are related to means.

Table 2 reports significant differences measured in fecal samples at a family and genus level. Families of *Clostridiaceae* (*p* = 0.002) and *Turicibacteraceae* (*p* = 0.027), belonging to the Firmicutes phylum, were significantly different between groups, showing a higher abundance in feces belonging to the R group in comparison to the G and Y group. Instead, *Peptostreptococcaceae* and *Ruminococcaceae* taxa, phylum Firmicutes, showed a trend with a *p*-value below to 0.1, where the former had a tendency for higher concentration in fecal samples belonging to the R group and the latter showed a tendency for lower concentration in the same samples. The genera *Clostridium* (*p* = 0.012), *SMB53* (family *Clostridiaceae*; *p* = 0.041)) and *Turicibacter* (family *Turicibacteriaceae*; *p* = 0.027) were significantly higher in R group. Genus *Dorea*, belonging to the *Clostridiaceae* family, was significantly lower (*p* = 0.041) in subclinical mastitis classified cows (R group) in comparison to the other two groups. Bacteria belonging to genus *Epulopiscium* showed a trend with a *p*-value below 0.1 and was found to be higher in the R group.

Table 3 reported taxa statistically different at a family and genus level in blood samples of cows classified in the three groups. Bacteria of the *Mycoplasma* genus (phylum Tenericutes) were significantly different (*p* = 0.020) between the three groups, with the highest abundance on cows with mastitis (group R) and the lowest one in healthy cows (group G). *Corynebacterium* (phylum Actinobacteria) highlighted a trend with a *p*-value below 0.1 and the highest content was found in samples belonging to the Y group.

In milk samples, several bacterial taxa differences between groups (Table 4) were observed, although most of them showed just a trend (*p*-value < 0.1). Proteobacteria was the phylum predominant in the milk, having the highest concentration in group Y and the lowest in the R group (*p* = 0.033). OD1 phylum also was significant (*p* = 0.002), having a higher concentration in milk samples of the R group. At the family level, bacteria belonging to *Enterobacteriaceae* (*p* = 0.043) and *Nocardiaceae* (*p* = 0.017) taxa were found to be statistically different between the three groups of animals. Both families were higher in animals of the R group in comparison to those of the G group, which showed intermediate abundances, and those of the Y group, with the lowest abundance. At the genera taxonomic level, of note was *Rhodococcus* (phylum Actinobacteria, family *Nocardiaceae*), which was significantly different between groups (*p* = 0.017), with the highest concentration in subclinical animals (group Y).

**Figure 2:**
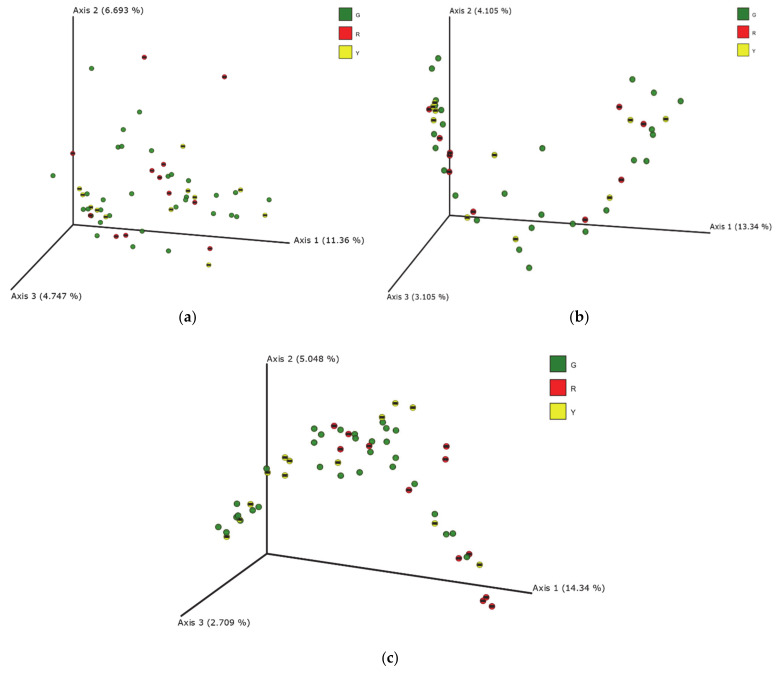
Principal coordinates analysis (PCoA) of the unweighted UNIFRAC distances of the microbial populations measured in feces (**a**), blood (**b**) and milk (**c**) sampled from lactating cows classified as healthy (G), at risk (Y) and with mastitis (R) on the basis of total somatic cell count (SCC) and differential somatic cell count (DSCC). PERMANOVA confirmed the differences between the G, Y and R groups of cows at *p* < 0.05 in milk samples. G = healthy, SCC/mL < 200,000 and DSCC ≤ 69.3%; Y = at risk, SCC/mL < 200,000 and DSCC > 69.3%; R = mastitis, SCC/mL > 200,000.

Beta biodiversity index calculated on the unweighted UniFrac distance matrices for feces, blood and milk are reported in Figure 2 as PCoA. Significant differences between the three experimental groups (G, Y and R) were calculated in milk samples (Figure 2c). PERMANOVA analysis of these data revealed significant differences between the G and R groups (*p* = 0.004) and between the Y and R groups (*p* = 0.006). Moreover, the R group presented the lowest richness in comparison to the other two experimental groups. The result of PERMANOVA analysis in milk microbiota is reported in Table S2.

**Figure 3:**
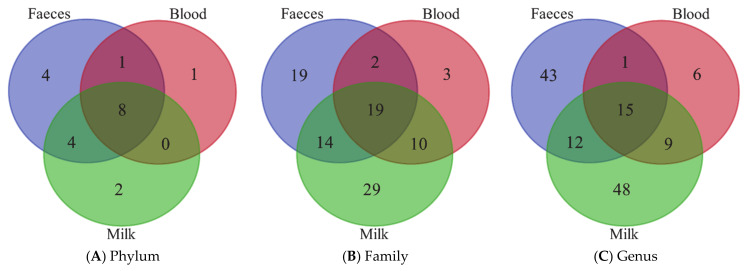
Venn diagrams showing the phyla (**A**), families (**B**) and genus (**C**) shared between milk, blood and milk samples from the lactating cows.

Venn diagrams (Figure 3) report the results of the annotation comparisons between feces, blood and milk at three different taxa levels, phylum, family and genus. As expected, the amounts of bacteria in the blood (Table 3) and milk (Table 4) were very low in comparison to feces (Table 2). The number of the resulted annotated taxa was very high in each biological matrix, although the microbial abundances were very low in blood and milk. Nevertheless, feces, blood and milk microbiomes shared 8, 19 and 15 taxa annotated at a phylum, family and genus level, respectively. The annotated taxa shared by the three matrices are reported in Table S3.

Correlations were calculated for common taxa between feces and blood, blood and milk and feces and milk at phylum, family and genera levels. Only OD1 phylum in feces and blood (r = −0.307; *p* < 0.05) and Bacteroidetes phylum in milk and blood (r = 0.298; *p* < 0.05) resulted in being significantly correlated. Coefficients of correlation were also computed between taxa and SCC and DSCC; no significant results were obtained. The inconsistent relationship observed did not allow further speculation. Due to the poor information given by the results of these correlations, data are not shown.

## 4. Discussion

Bovine mastitis is a pathology caused by the inflammation and the infection of the mammary gland and leads to important economic losses each year in the dairy industry all over the world, as well as negatively impacting animal wellbeing [38]. This disease is caused by several different pathogens, including some with an environmental reservoir, such as environmental streptococci (i.e., *S. uberis* and *S. dysgalactiae*) and coliforms (*Escherichia coli* and *Klebsiella* spp.) [39]. These bacteria are commonly present in the stable environment; thus, the findings of microbial population in milk has been always considered a transgression of environmental microbial contaminants through the teat canal (TC) [40]. Recently, the hypothesis that bacteria cross from the gut to the milk, passing though the blood, has been the object of some research. This so-called entero-mammary pathway (EMP) was also supported by experiments in mice and humans [3].

In the present study, the presence of bacterial DNA in the feces, blood and milk of healthy, at risk and chronically mastitic animals was analyzed and several taxa were detected. As shown in Figure 1, the majority of the phyla annotated in the three matrices were shared between each other, although with different relative abundances.

The fecal microbiome of bovines is characterized by four predominant phyla. Based on the study of Kim and Wells [41], Firmicutes is the most represented phylum, with Clostridia and *Faecalibacterium* as the largest class and genus, respectively. The second most represented phylum is Baceroidetes, with Bacteroidia as the largest class and *Prevotella* as the largest genus. Proteobacteria is another well-represented phylum, that has Gammaproteobacteria and *Succinivibrio* as the most abundant class and genus, respectively. From the study of Cendron et al. [42], Actinobacteria phylum is also a large group of bacteria presents in the fecal microbioma of cows. These four phyla reported for the fecal microbiota (Firmicutes, Proteobacteria, Bacteroidetes, and Actinobacteria) constitute the main bacteria groups shaping the structure of milk microbiota [43,44,45]. Our results agreed with the microbial composition in feces and milk already reported in previous research [43,44,45]. Limited information is reported for blood and some evidence of the presence of blood microbiome exists in various domesticated animals, such as mammals and birds [18,19] and in humans [20,21,22].

Gut microbiota in ruminants is composed by hundreds of bacteria species, prevalently from phylum Firmicutes, Bacteroidetes, Actinobacteria and Proteobacteria [46], which have considerable abundances and shift in relation to diet, genetic and environmental factors such as high temperatures [46,47]. The taxa significantly different between groups were limited to the family of *Clostridiaceae* and *Turicibacteriaceae* and to the genera of *Clostridium*, *Dorea*, *SMB53* and *Turicibacter*. The majority of study that characterized the gut microbiome of cows regards the role of the rumen microbiota in gastrointestinal health and for the feed efficiency [47,48]. Some differences in terms of microbiota composition between healthy (G group), at risk (Y group) and mastitic (R group) cows were expected, and some significant variations were found between groups, but not as high as expected given the health conditions of the cows at the time of sampling. The limited knowledge about the intestinal microbiome of cows and the relation with inflammatory diseases, mastitis in particular, makes it difficult to explain the variation in taxa observed in this study.

Blood was considered a sterile environment, and the nonpathogenic condition of the blood microbial population can be explained as the “dormant phase” of bacteria [49]. However, occasionally, some of these bacteria could start to reproduce again and to become active again. From the blood, some of the bacteria can be transported to peripheral tissues and organs, inducing chronic disease [50].

In blood, the bacteria that distinguished the groups of animals were not those found in the feces and only the genus *Mycoplasma*, of the phylum Tenericutes, was significantly higher for the R group in comparison to the other groups. The Mycoplasma species cause severe disease in dairy cows and are responsible for mastitis and other diseases. The *Mycoplasma* genus is mainly transmitted during milking and is also infrequent in Italian dairy herds [28]. However, there are studies that support the idea that *Mycoplasma* spp. can be spread from blood to the mammary gland to cause mastitis and arthritis [51]. In this study, this genus was higher in blood samples from the Y and R groups, but it was not recovered from milk samples, supporting the evidence that *Mycoplasma* spp. may spread through blood stream, but this way has little importance in the development of udder infections. In milk, the differences between groups were limited and of interest for the Gram-negative family of *Enterobacteriaceae*, which is typically present in the gut and includes several pathogens, such as *Salmonella*, *Escherichia* and *Shigella* [52]. With 16S rRNA, the annotation at species level is not robust enough and other methodologies are indicated to identify the specie and the strain of pathogens. Indeed, the results obtained from this study paved the way to consider mastitis from a different point of view, such as the study of the microbial community and its biodiversity in relation to the healthy conditions of the mammary gland. The microbiome can also play a paramount role in determining milk quality, either as a natural probiotic [53,54] or for the improvement of dairy processes [55,56].

The microbial diversity in milk revealed differences between the cows grouped on the basis of SCC and DSCC, as confirmed by PCoA analysis (Figure 2). Indeed, beta diversity was not affected by the udder status, in feces and blood. The clustering observed in milk samples was confirmed by the PERMANOVA analysis, highlighting the differences between the G, Y and R groups of cows (*p* < 0.05). Our results are not consistent with those reported by Taponen et al. [45], which did not observe significant differences of microbiome in milk sampled in infected and non-infected quarters of the same cow. Unexpectedly, the greatest differences were observed between the Y and R groups, whereas intermediate values were reported for the G group. It is likely that cows at risk of mastitis (Y group) mount an inflammation response that modulates the bacterial community stronger in comparison to a chronic condition. However, considering that few studies on the microbiome composition of milk from cows with mastitis are published, further research is needed to clarify the modifications that inflammatory conditions and microbial invasion can cause on bacterial population.

The Venn diagrams (Figure 3) showed a high number of taxa in common between feces, blood and milk, at a phylum, family and genus level. The so-called “leaky gut” condition is widely studied in humans and dogs and often is related to inflammatory bowel diseases and related enteropathies [57,58,59,60], but it is not reported yet if other pathological conditions, metabolic imbalance or stressful events can alter intestinal barrier integrity. Although the presence of the same taxa was found in all three specimens, it is hard to confirm that the translocation of microbiome from one site to another occurred and that this was a concurrent cause of mastitis. External factors, such as contamination with environmental bacteria during the sampling, could have led to an artifactual appearance of microbiome into the blood and milk. For what the reagents and the sequencing pipeline is concerned, a mock bacterial community was used as an internal standard to validate the methodology, and the results confirmed the lack of contamination.

## 5. Conclusions

Considering the results of this study, it is not possible to confirm a bacterial crossing from the gut to milk in cows, that still remain hypothetical. The investigation of the entero-mammary pathways in ruminants still deserves further study. To confirm the translocation, the analysis of microbiota whole genomes and the presence of the same bacteria in blood versus feces and milk could help to unravel the process behind the entero-mammary pathway. Minor differences were found for the composition of the microbiome in feces, blood and milk between the groups of cows but the beta diversity indicated that microbial communities of milk changes in relation to the healthy condition of the cow.

## Figures and Tables

**Table 1:** Partitioning of lactating cows in healthy (G), at risk (Y) and with mastitis (R) on the basis of total somatic cell count (SCC) and differential somatic cell count (DSCC).

Group	Number of Cows	Age (Parturitions)		DIM	
		Primiparous	Pluriparous	<70	>70
G	34	11	23	10	24
Y	13	6	7	2	11
R	13	2	11	3	10

**Table 2:** Statistical comparison of the mean abundances of bacteria at a family and genus taxonomic levels measured in feces sampled from lactating cows classified as healthy (G), at risk (Y) and with subclinical mastitis (R) on the basis of total somatic cell count (SCC) and differential somatic cell count (DSCC). Only taxa which significantly differed with the Kruskal–Wallis non-parametric test are reported.

Taxa	G (AA)			Y (AA)			R (AA)			
	Mean		SD	Mean		SD	Mean		SD	*p*-Value
**Family**										
Clostridiaceae	21.8	^a^	10.1	18.6	^a^	8.0	31.6	^b^	11.4	0.002
Peptostreptococcaceae	28.7		15.3	24.8		13.0	35.9		14.8	0.077
Ruminococcaceae	214.8		56.5	213.9		59.6	194.5		60.5	0.077
Turicibacteraceae	16.2	^ab^	9.0	13.1	^a^	8.0	22.4	^b^	10.5	0.027
**Genera**										
*Clostridium*	42.7	^a^	20.5	36.4	^a^	17.1	56.3	^b^	19.8	0.012
*Dorea*	7.2	^ab^	2.9	8.0	^b^	4.0	5.8	^a^	2.2	0.040
*Epulopiscium*	1.7		1.3	1.5		1.3	2.5		1.4	0.060
*SMB53*	0.3	^ab^	0.2	0.2	^a^	0.2	0.4	^b^	0.3	0.041
*Turicibacter*	16.2	^ab^	9.0	13.1	^a^	8.0	22.4	^b^	10.5	0.027

AA = absolute abundances, number of DNA copies; G = healthy, SCC/mL < 200,000 and DSCC ≤ 69.3%; Y = at risk, SCC/mL < 200,000 and DSCC > 69.3%; R = mastitis, SCC/mL > 200,000. ^a,b^ on the same row denotes differences between means for *p*-value < 0.05.

**Table 3:** Statistical comparison of the mean abundances of bacteria at a phylum and genus taxonomic levels measured in blood sampled from lactating cows classified as healthy (G), at risk (Y) and with subclinical mastitis (R) on the basis of total somatic cell count (SCC) and differential somatic cell count (DSCC). Only taxa which significantly differed with the Kruskal–Wallis non-parametric test are reported.

Taxa	G (AA)			Y (AA)			R (AA)			
	Mean		SD	Mean		SD	Mean		SD	*p*-Value
**Phylum**										
Tenericutes	0.6	^ab^	1.2	1.1	^b^	1.2	1.4	^a^	3.8	0.020
**Genera**										
*Corynebacterium*	0.1		0.2	0.5		0.8	0.2		0.2	0.097
*Mycoplasma*	0.6	^b^	1.2	1.1	^ab^	1.2	1.4	^a^	3.8	0.02

AA = absolute abundances; G = healthy, SCC/mL < 200,000 and DSCC ≤ 69.3%; Y = at risk, SCC/mL < 200,000 and DSCC > 69.3%; R = mastitis, SCC/mL > 200,000. ^a,b^ on the same row denotes differences between means for *p*-value < 0.05.

**Table 4:** Statistical comparison of the mean abundances of bacteria at a phylum, family and genus taxonomic levels measured in milk sampled from lactating cows classified as healthy (G), at risk (Y) and with subclinical mastitis (R) on the basis of total somatic cell count (SCC) and differential somatic cell count (DSCC). Only taxa which significantly differed with the Kruskal–Wallis non-parametric test are reported.

Taxa	G (AA)			Y (AA)			R (AA)			
	Mean		SD	Mean		SD	Mean		SD	*p*-Value
**Phylum**										
Actinobacteria	28.6		18.6	29.5		15.5	22.1		21.2	0.099
OD1	0.2	^a^	0.2	0.1	^a^	0.2	0.4	^b^	0.4	0.002
Proteobacteria	106.5	^ab^	63.4	170.6	^b^	98.8	90.9	^a^	54.8	0.033
**Family**										
Cellulomonadaceae	0.3		0.5	1.0		1.7	0.1		0.2	0.074
Enterobacteriaceae	1.2	^ab^	2.5	5.2	^b^	13.6	0.3	^a^	0.5	0.043
Microbacteriaceae	10.7		8.6	13.2		8.7	7.5		8.4	0.091
Moraxellaceae	54.0		52.5	115.7		95.4	57.9		52.6	0.054
Nocardiaceae	1.2	^b^	1.3	1.7	^b^	1.9	0.7	^a^	1.3	0.017
Phyllobacteriaceae	0.5		1.4	0.6		0.9	0.0		0.1	0.092
Propionibacteriaceae	2.8		3.3	4.4		7.0	1.3		1.6	0.076
Pseudomonadaceae	5.2		7.4	7.0		7.0	2.3		3.9	0.063
Succinivibrionaceae	0.0		0.1	0.0		0.0	0.0		0.1	0.085
Williamsiaceae	0.1		0.2	0.1		0.1	0.0		0.1	0.077
**Genera**										
5-7N15	0.2		0.2	0.1		0.1	0.2		0.2	0.065
*Acinetobacter*	51.3		51.2	110.1		92.2	54.8		50.9	0.055
*Aminobacter*	0.5		1.4	0.6		0.9	0.0		0.1	0.092
CF231	0.1		0.2	0.0		0.1	0.1		0.1	0.087
*Microbacterium*	4.8		4.0	5.3		3.9	2.4		2.6	0.052
*Olsenella*	0.0	^ab^	0.0	0.0	^b^	0.1	0.0	^a^	0.0	0.044
*Propionibacterium*	2.8		3.3	4.4		7.0	1.2		1.6	0.056
*Pseudomonas*	5.1		7.4	6.9		6.8	2.3		3.9	0.055
*Rhodococcus*	1.2	^ab^	1.3	1.7	^b^	1.9	0.7	^a^	1.3	0.017
*Ruminobacter*	0.0		0.1	0.0		0.0	0.0		0.1	0.085
*Staphylococcus*	0.2		0.4	0.5		0.6	0.8		2.1	0.098
*Williamsia*	0.1		0.2	0.1		0.1	0.0		0.1	0.077

AA = absolute abundances; G = healthy, SCC/mL < 200,000 and DSCC ≤ 69.3%; Y = at risk, SCC/mL < 200,000 and DSCC > 69.3%; R = mastitis, SCC/mL > 200,000. ^a,b^ on the same row denotes differences between means for *p*-value < 0.05.

## Data Availability

The data presented in this study are openly available in NCBI Sequence Read Archive (Bioproject ID: PRJNA725200).

## Supplementary Materials

The following are available online at https://www.mdpi.com/article/10.3390/ani11051463/s1, Table S1: Bacteria shared by the three biological matrices analyzed in this study; Table S2: PERMANOVA results based on unweighted UniFrac distance on milk microbiome; Table S3: Bacteria shared by the three biological matrices analysed in this study
